# Assessment of Liver Antioxidant Profile in *Plasmodium Berghei* Infected Mice Treated with Curative Ethanol Leaf extract of *Musa paradisiaca*

**DOI:** 10.4314/ejhs.v33i5.6

**Published:** 2023-09

**Authors:** Maureen Ibitoroko George-Opuda, Olugbenga Adebayo Adegoke, Bensandy Othuke Odeghe, Abimbola Temitayo Awopeju, Ngozi Margret Okeahialam

**Affiliations:** 1 Department of Medical Laboratory Science, Rivers State University, Port Harcourt, Nigeria; 2 Department of Medical Laboratory Science, Madonna University, Elele, Nigeria; 3 Department of Medical Biochemistry, Faculty of Basic Medical Sciences, Delta State University, Abraka, Nigeria; 4 Department of Medical Microbiology, College of Medicine, University of Port Harcourt, Nigeria

**Keywords:** Musa paradisiaca, Plasmodium berghei, liver, antioxidant, curative

## Abstract

**Background:**

The increasing resistance to most antimalarial drugs suggests a need for better alternatives. This study evaluated in vivo antimalarial and liver antioxidant profile of dry plantain leaf extract (Musa paradisiaca) on mice infected with Plasmodium berghei.

**Methods:**

Six groups of ten mice each grouped as control, P. berghei, artesunate, and P. berghei infected mice were orally administered 250,500 and 1000mg/kg Musa paradisiaca leaf extract for 5 days. Blood smears were evaluated for parasitaemia on the 10^th^ day and the mice sacrificed. Catalase, Malondialdehyde, protein, Glutathione peroxidase and reduced glutathione was estimated using Colorimetric, Biuret and spectrophotometric methods respectively with data analyzed using SPSS version 21.

**Results:**

Catalase activity (umol/ml/mins) was 24.62 ± 0.99, 10.04 ± 0.50, 19.35 ± 0.38, 22.13 ± 0.00, 22.79 ± 0.00 and 23.66 ± 0.20 while Glutathione Peroxidase(u/l) was 332.34± 0.64, 205.22± 4.61, 218.26± 0.63, 310.59± 0.00, 305.20± 0.00. and 295.97± 0.02 at Control, P.berghei, artesunate, 250mg, 500mg and1000mg extracts. Glutathione (mM) was 1.60 ± 0.12, 0.64 ± 0.09, 1.06 ± 0.16, 0.72 ± 0.00, 0.92 ± 0.00 and 1.26 ± 0.08 while Malondialdehye (uM) was 16.93 ± 3.59, 61.65 ± 1.72, 27.80 ± 0.26, 36.90 ± 0.00, 34.30 ± 0.00 and 32.68 ± 0.27 and Protein(g/dl) was 22.37 ± 1.87, 7.91 ± 0.13, 11.78 ± 1.19, 11.79 ± 0.00, 13.20 ± 0.00 and 17.04 ±0.03 at control, P.berghei, artesunate, 250mg, 500mg and1000mg respectively.

**Conclusion:**

The study suggested that ethanolic extract of Musa paradisiaca reduced liver oxidative stress caused by P.berghei.

## Introduction

*Plasmodium berghei berghei* is a species in the genus *Plasmodium subjenus Vinckeia* (1). It is a protozoan parasite that causes malaria in certain rodents. Originally, isolated from thicket rats in Central Africa, *P. berghei* is one of four plasmodium species that have been described in African murine rodents, the others *Plasmodium chabaudi, Plasmodium vinckei*, and *Plasmodium yoelii*. Due to its ability to infect rodents and relative ease of genetic engineering, *P. berghei* is a popular model organism for the study of human malaria. The rodent parasite *Plasmodium berghei* is one of the well-employed models in malaria related research, and this includes analysis on severe pathology associated with malaria infections. It has been reported that *Plasmodium berghei* can induce a number of disease states in rodents (1).

*Musa paradisiaca* is a monoherbacious plant belonging to the family Musaceae, distributed throughout the tropical and subtropical countries. The plant parts are widely used to treat different diseases in humans in traditional medicines, such as diabetes, diarrhea, dysentery, hypertension, hysteria, epilepsy, leprosy, hemorrhages, renal calculi and ulcers. The main pharmacological activities of this plant are antilithiatic, antioxidant, antibacterial, antidiabetic, antiulcer, antidiarrhoeal, hypocholesterolaemic, hepatoprotective, antisnakevenom, wound healing, hair growth promoting, and antifungal and antimenorrhagic activity. A review by Lavanya *et al.*, (2) presented information on morphology, traditional uses, phytochemistry, and pharmacological activities of *Musa paradisiaca*.

Antioxidants are molecules that in low concentrations can prevent or delay the oxidation of an oxidizable substrate (3). Antioxidants are present in our body and exist in several foods. Antioxidants have a high affinity for FR and scavenge these molecules to protect our health. Compounds with antioxidant properties donate electrons to FR to reduce their reactivity and maintain cellular pro-oxidant/oxidant balance. There are many types of molecules with antioxidant activity. Some selected liver antioxidants include Catalase (CAT), Glutathione peroxidase (gpx), Protein, Malondialdehyde (MDA), and Reduced Glutathione (GSH). The aim of this study is to assess liver antioxidant profile of *Plasmodium berghei* infected mice treated with ethanol leaf extract of *Musa paradisiaca*

## Materials and Methods

**Study period, area and design**: This experimental study using mice was carried out between the months of January to June 2022 at Madonna University. Madonna University, the first Catholic University in Nigeria, was founded by Very Rev. Fr. Professor E.M.P Edeh with three campuses in Elele, Akpugo and Okija in 1999 (4).The University is a center for academic and research excellence. This study was carried out at the Elele campus of the University which has the state-of-art research facility and a Medical Laboratory services attached to the Teaching Hospital.

**Animal**: A total of 84 healthy albino mice of about 2-3 months old and weighing between 13-36g obtained from animal house of the Federal University of Technology, Owerri, Nigeria, were used for the study. They were kept in the animal house of Madonna University, Elele campus, and were housed in aluminum cages at room temperature and under light/darkness cycles. The mice were supplied with clean drinking water and fed *ad libitum* with standard commercial pelleted starter feed (vital feed) and were acclimatized for two weeks before administration. They were maintained in accordance with their commendations of the guide for the care and use of laboratory animals and experimental protocol was approved by the institution.

**Extraction and preparation of plant materials**: The leaves of *Musa paradisiaca* were obtained from a plantain plantation in Elele, Rivers State, Nigeria. They were cut into pieces, washed and dried. The dried pieces were grounded into fine powder using a manual grinder. Ten kilogram (10kg) of the grounded powder was soaked in 10litres of 80% ethanol for 72 hours with intermittent stirring of the solution. The mixture was subsequently filtered through Whatman filter paper (125mm). The extract was concentrated using a rotary evaporator at 45°C and then dried with a water bath at 39°C to yield 40g of dark semi-solid extract and kept at 0-4°C until needed for use.

**Parasite inoculation**: A chloroquine sensitive strain of *Plasmodium berghei* (NK 65 strain) parasite used in this study was obtained from the Department of Pharmacology and Toxicology, Faculty of Basic Clinical Sciences, University of Port Harcourt, Rivers State, Nigeria. The *Plasmodium berghei* was sustained by constant re-infestation of parasitized erythrocytes which were sourced from a donor-infected mouse by the tail via a heparinized syringe and made up to 20ml with normal saline. The animals were inoculated with 0.2ml of infected blood suspension. The donor mice were monitored for signs of infection which include lethargy, anorexia, shivering and heat-seeking environment. Parasitemia was monitored daily by microscopic examination of Giesma stained thick film and viewed at × 100 objective. Furthermore, malaria parasite infection was detected by thin film stained with Leishman stain by parasite count using the formula: Parasite density =Number of parasites counted × 100white blood cells/Number of white blood cells counted.

**Acute toxicity study** **(LD_50_)**: The crude extract of *Musa paradisiaca* leaf was evaluated for their toxicity in *P. berghei* non-infected Swiss albino mice aged 2 months and weighing 18-20g using, modified Locke's (5) method of determining toxicity level of extract in mice. The test was carried out in two phases. In the phase one of the study, twelve mice were randomized into three groups of four mice each and were given (500, 1000 and 3000) mg/kg body weight respectively of the extract orally. The mice were observed for changes in physical appearance, gross behavioral change and death in the first four hours and subsequently daily for five days. In view of the result obtained from phase I treatment, phase II treatment was carried out using another fresh set of twelve mice randomized into three groups of four mice each and were given (500, 1000 and 3000) mg/kg body weight of the extract orally. These were observed for signs of toxicity and mortality for the first four hours and thereafter daily for ten days (5). The LD_50_ was then calculated as the square root of the product of the lowest lethal dose and highest non-lethal dose, i.e., the geometric mean of the consecutive doses for which 0 and 100% survival rates were recorded in the second phase. The oral median lethal dose was calculated using the formula: LD_50_ =√maximum dose for all survival × minimum dose for all death (5).

**Experimental design using Curative (Rane Test) by evaluating Schizontocidal activity in Established Infection**: The curative potential evaluation of the extract was done using a method of Ryley and Peter (6). A total of 60 male and female mice weighing 10-37g were assigned into six groups of ten mice each; namely, control (A), *P. berghei* (B), artesunate (C) , 250(D), 500(E), and 1000mg/kg/day(F). The group A received strictly feed and water adlibitum to serve as control mice throughout the study period while the groups B,C,D,E, and F mice were injected with standard inoculums of 1x10^7^ *P. berghei* infected erythrocytes intraperitoneally on the first day (Day 0). Seventy-two hours later (day 3), the mice in groups C,D,E, and F mice were orally administered with 50mg/kg artesunate, *Musa paradisiaca leaf* extract of 250, 500 and 1000mg/kg/day, respectively. The drug extract was given orally once daily for 5 days. Thin films stained with 10% Giemsa at pH 7.2 for 10 minutes and parasitaemia were examined microscopically on each day of treatment to monitor parasiteamia level.

**Sample collection and analysis**: At the end of 10 days of treatment, the blood samples were collected with a sterile syringe and needle and was put in plain container bottle. The samples were centrifuged at 1500rpm (revolution per minute) for 10 minutes. After centrifugation, the serum obtained was used for the analysis.

**Catalase estimation**: Catalase estimation was done according to method of Mahmoud and Haider (7) using spectrophotometric method. This assay method is based on the principle of the measurement of the hydrogen peroxide substrate remaining after the action of catalase. First, the catalase converts hydrogen peroxide to water and oxygen (catalytic pathway), and then, this enzymatic reaction is stopped with sodium azide. An aliquot of the reaction mix is then assayed for the amount of hydrogen peroxide remaining by a colorimetric method. The colorimetric method uses a substituted phenol (3,5-dichloro-2-hydroxybenzenesulfonic acid), which couples oxidatively to 4-aminoantipyrine in the presence of hydrogen peroxide and horseradish peroxidase (HRP) to give red quinoneimine dye (N-(4-antipyryl)-3-chloro-5-sulfonatep-benzoquinone monoimine) that absorbs at 520 nm.

***Procedure***: The assay reaction was performed at room temperature (25 °C). The Assay Buffer, Colorimetric Assay Substrate Solution, and Color Reagent were allowed to equilibrate to room temperature. Into a test tube, 25ul of sample and 75ul of assay buffer were dispensed, then the reaction was started by addition of 25ul of substrate B to the test tube. It was mixed by inversion and incubated at 25◦c for 15 minutes. Eight hundred and twenty five microliter (825ul) of the stop solution was added and mixed. Ten microliter (10ul) aliquot of the mixture was removed and added to another test tube. One milliliter (1ml) of chromogen reagent was added and mixed well. Then, it was allowed to stand for 15 minutes at room temperature to develop color. Change in absorbance was read at 520nm wavelength.

**Glutathione peroxidase (GPX) estimation**: GPX estimation was carried out according to method of Charmagnol *et al.*, (8) as modified by Sigma Aldrich diagnostic using Spectrophotometric method. The **Principle showed that** Glutathione peroxidase catalyses the oxidation of Glutathione (GSH) by cumene hydroperoxides. The oxidized glutathione is converted to the reduced form in the presence of glutathione reductase and NADPH. In this reaction, the NADPH is oxidized to NADP^+^ simultaneously. The decrease in absorbance at 340nm is then measured.

***Procedure***: Into a clean microcuvette 20ul of sample was added and 20µl of distilled water into another cuvette (Reagent Blank), then 1ml of working reagent was added to each cuvette. Forty microlitre (40ul) of cumene hydroperoxide solution was added to each cuvette. It was mixed and initial absorbances of sample and reagent blank were read after 1 minute, and timer was started simultaneously. It was read again after 1 and 2 minutes. The reagent blank value was subtracted from that of the sample.

**Protein estimation**: Protein estimation was done by Biuret method according to Henry *et al.*, (9) as modified by Fortress diagnostic. Copper ions react in alkaline solution with protein peptide bonds to give a purple coloured biuret complex. The amount of complex formed is directly proportional to the amount of protein in the specimen.

***Procedure***: Into three test tubes labelled test, standard, and blank, 1000ul of biuret reagent was added to all. Twenty microlitre (20ul) test sample were added to test, 20ul of standard was added to standard and 20ul of distilled water was added to blank. The three test tubes containing the solutions were mixed thoroughly and incubated for 10 minutes at 37^O^C. Their absorbance was read spectrophotometrically at 546nm wavelength.

**Malondialdehyde (MDA) estimation**: MDA estimation was done by Colorimetric method. The **Principle of** this assay is based on the reaction of a chromogenic reagent, 2-thiobarbituric acid, with MDA at 25°C. One molecule of MDA reacts with 2 molecules of 2-thiobarbituric acid via a Knoevenagel-type condensation to yield a chromophore with absorbance maximum at 532 nm.

***Procedure***: The Free MDA and Total MDA were estimated as shown below.

***Free MDA***: Into glass tubes labelled standards, samples and blank, 200ul of standard, sample and 200ul of indicator solution were added. Two hundred microliter (200ul) of indicator solution were added respectively and mixed well. The mixture were allowed to react for 45 minutes at room temperature. Then, 300µl was transferred to a microplate, and the absorbance of the resulting solution was measured at 532 nm. The pink color is stable for several hours at room temperature.

***Total MDA***: Into glass tubes labelled standards, samples and blank, 200ul of standard, sample and indicator solution were added. Two hundred microliter (200ul) of indicator solution were added respectively and mixed well. The content of sample tube was heated at 65°C for 45 minutes. Three hundred microlitre (300µl) was transferred to a microplate and the absorbance was measured at 532 nm.

**Reduced glutathione (GSH) estimation**: GSH estimation was done by Spectrophotometric method using Ellman reagent (10) as modified by Sigma Aldrich diagnostic. These spectrophotometric procedures are based on the method of Ellman (10), who reported that 5,5′-dithiobis- (2-nitrobenzoic acid) is reduced by SH groups to form 1 mole of 2-nitro-5-mercaptobenzoic acid per mole of SH. The nitromercaptobenzoic acid anion has an intense yellow color which when measured at a wavelength of 412nm can be used to measure SH groups.

**Procedure:** Into a microcuvette, 100ul of standard and samples were dispensed respectively. Eight hundred and eighty microliter (880ul) of GSH dilution buffer was added to each microcuvette. Twenty microliter (20ul) of GSH chromogen was added to the microcuvette respectively and mixed. The absorbance of resulting solution was measured at 412nm wavelength within 5 minutes.

**Statistical analysis**: Data obtained were subjected to statistical analysis using Statistical Package for Social Science version 21 using statistical tools such as t-test and analysis of variance (ANOVA). Results were expressed as Mean ± Standard Deviation (X±SD). The values of P<0.05 were considered significant.

## Results

**Liver antioxidant profile in P.berghei infected mice treated with ethanol leaf extract of Musa paradisiaca**: The catalase activity (umol/ml/mins) of 24.62 ± 0.99 in control was reduced by *P.berghei* to 10.04 ± 0.50 which was significantly increased to 22.13 ± 0.00, 22.79 ± 0.00 and 23.66 ± 0.20 by 250mg, 500mg and 1000mg extract respectively. The glutathione peroxidase activity (u/l) of 332.34± 0.64 in control was reduced by *P.berghei* to205.22± 4.61 while administration of 250mg,500mg and 1000mg extract increased it to 310.59± 0.00, 305.20± 0.00 and 295.97± 0.02 respectively. The glutathione (mM) of 1.60 ± 0.12 in control was reduced by *P.berghei* to 0.64 ± 0.09 while administration of 250mg,500mg and 1000mg extract increased it to 0.72 ± 0.00, 0.92 ± 0.00 and 1.26 ± 0.08 respectively. The Malondialdehye (uM) of 16.93 ± 3.59 in control was increased to 61.65 ± 1.72 by *P.berghei* while administration of 250mg,500mg and 1000mg extract reduced it to 36.90 ± 0.00, 34.30 ± 0.00 and 32.68 ± 0.27 respectively. The protein (g/dl) concentration of 22.37 ± 1.87 was reduced by *P.berghei* to7.91 ± 0.13 while administration of 250mg, 500mg and 1000mg extract increased it to11.79 ± 0.00, 13.20± 0.00 and 17.04 ±0.03 respectively.

**Liver antioxidant profile in P.berghei infected mice treated with ethanol leaf extract of Musa paradisiaca**: The catalase activity (umol/ml/mins) of 24.62 ± 0.99 in control was reduced by *P.berghei* to 10.04 ± 0.50 which was significantly increased to 68.58±0.20 extract. The glutathione peroxidase activity (u/l) of 332.34± 0.64 in control was reduced by *P.berghei* to 205.22± 4.61 while administration of extract increased it to 911.76±0.02. The glutathione (mM) of 1.60 ± 0.12 in control was reduced by *P.berghei* to 0.64 ± 0.09 while administration of extract increased it to 2.90±0.08. The Malondialdehye (uM) of 16.93 ± 3.59 in control was increased to 61.65 ± 1.72 by *P.berghei* while administration of extract increased it to 103.88±0.27. The Protein (g/dl) concentration of 22.37 ± 1.87 was reduced by *P.berghei* to 7.91 ± 0.13 while administration of extract increased it to 42.03±0.03

## Discussion

Malaria still remains an important health issue in most African countries with respect to the number of people affected, levels of morbidity, and mortality (11), while in the Hana and Keyafer health centers located in South OmoZone, Southern Ethiopia, malaria has been reported as a public health problem (12). Exploring new plant-derived antimalarial drugs has increased after the success of artemisinin. However, reported and documented cases of resistance to these drugs including artemisinin made the research and development of new antimalarial drugs necessary (13). Several researchers have dedicated efforts to the development of new active compounds, especially from artemisinin (14), as an alternative to chloroquine (15). Unfortunately, first reports on drug resistance to artemisinin-derivatives (16) and to drug combination therapies (13) have already appeared. There is a consensus among the scientific community that natural products have been playing a dominant role in the discovery of leads for the development of drugs for the treatment of human diseases (17). Increased use of herbal plants and formulations to treat malaria among other diseases has led to the need for scientific investigation and documentation of plants that possesses anti-plasmodia and/or antimalarial activities in a bid to validate the claims for their use in folklore (18). Plants are major sources of bioactive compounds with potential for developing new and original antimalarial drugs (19). Hence, this study focused on evaluating the antimalarial and the biochemical effect of *Musa paradisiaca* on selected liver antioxidants using albino mice.

The result of the study showed that *P.berghei* treatment caused reduced Catalase, Reduced glutathione, Glutathione peroxidase, and Protein with increased Malondialdehyde. This is suggestive that *P.berghei* treatment caused liver oxidative stress. Treatment of infected mice with curative doses of *Musa paradisiaca* extract and artesunate significantly (p<0.05) increased the Catalase, Reduced glutathione, Glutathione peroxidase and Protein while reducing Malondialdehyde, in the infected mice. These findings are in consonance with earlier reports on the rise in MDA levels indicative of lipid peroxidative in the liver (20). There was a significant (p<0.05) difference in the levels of Catalase (CAT), Malondialdehyde (MDA), Glutathione peroxidase (GPX), Reduced glutathione (GSH), and protein in mice administered 250, 500 and 1000 mg/kg body weight in all the curative model.

In this study, malaria parasite infection markedly induced a state of oxidative in host (mice) as indicated by significant decrease in Catalase, Glutathione peroxidase, Protein, Reduced glutathione levels and increase in parasitized non treated mice. The extract was effective in the reduction of biomarkers such as Catalase (CAT), Malondialdehyde (MDA), Glutathione peroxidase (GPX), Reduced glutathione (GSH), and protein. The study has shown that ethanolic extract of *Musa paradisiaca* has the tendency to reduce liver oxidative stress caused by *P.berghei*.

## Figures and Tables

**Table 1:** Liver antioxidant profile in *P.berghei* infected mice treated with ethanol leaf extract of *Musa paradisiaca*

Group	Catalase (umol/ml/mins)	Glutathione peroxidase (u/l)	Glutathione (mM)	Malondialdehye (uM)	PROTEIN (g/dl)
Control	24.62 ± 0.99	332.34± 0.64	1.60 ± 0.12	16.93 ± 3.59	22.37 ± 1.87
*P. berghei*	10.04 ± 0.50*	205.22± 4.61*	0.64 ± 0.09*	61.65 ± 1.72*	7.91 ± 0.13*
Treated					
Curative	19.35 ± 0.38**	218.26± 0.63*	1.06 ± 0.16**	27.80 ± 0.26*,**	11.78± 1.19*
artesunate					
250mg extract	22.13 ± 0.00**	310.59± 0.00**	0.72 ± 0.00*	36.90 ± 0.00*,**	11.79± 0.00*
500mg extract	22.79 ± 0.00**	305.20± 0.00**	0.92 ± 0.00*	34.30 ± 0.00*,**	13.20±0.00*,**
1000mg extract	23.66 ± 0.20**	295.97± 0.02**	1.26± 0.08**	32.68 ± 0.27*,**	17.04±0.03*,**
P	0.000	0.000	0.000	0.000	0.000

* Compare with the control

** Compare with the *P. berghei* treated
